# Multiple painless papulonodules in a 3-year-old girl: type A lymphomatoid papulosis^☆^

**DOI:** 10.1016/j.abd.2020.11.020

**Published:** 2022-07-13

**Authors:** Nuno Gomes, Ana Nogueira, Roberto Silva, Filomena Azevedo

**Affiliations:** a Department of Dermatovenereology of Centro Hospitalar, Universitário de São João EPE, Porto, Portugal; b Department of Pathology of Centro Hospitalar, Universitário de São João EPE, Porto, Portugal

Dear Editor,

A 3-year-old girl, otherwise healthy, presented with a dermatosis characterized by asymptomatic erythematoviolaceous papules and nodules with slight desquamation on the trunk and lower limbs, one of them with spontaneous ulceration, evolving for 3 months (Figure 1, Figure 2). She was in a good general condition and no lymph nodes were palpable. Her mother denied any previous infectious episodes. She had been applying a methylprednisolone aceponate cream for the previous 2 weeks without improvement. She had no significant past medical or family history. A skin biopsy of the lower right limb was performed (Fig. 3). A diagnosis of lymphomatoid papulosis was made.

**Figure 1:**
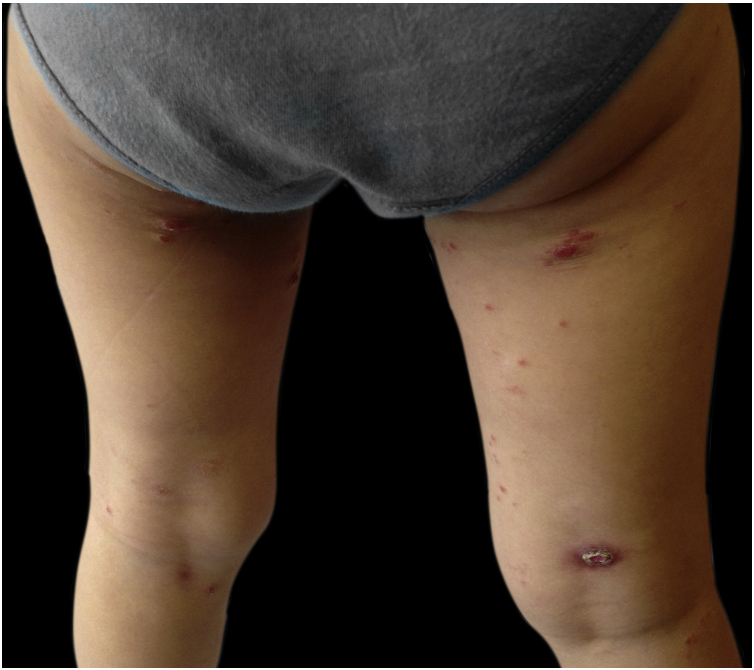
Erythematoviolaceous papules and nodules with slight desquamation on the lower limbs, one of them with spontaneous ulceration (right thigh).

**Figure 2:**
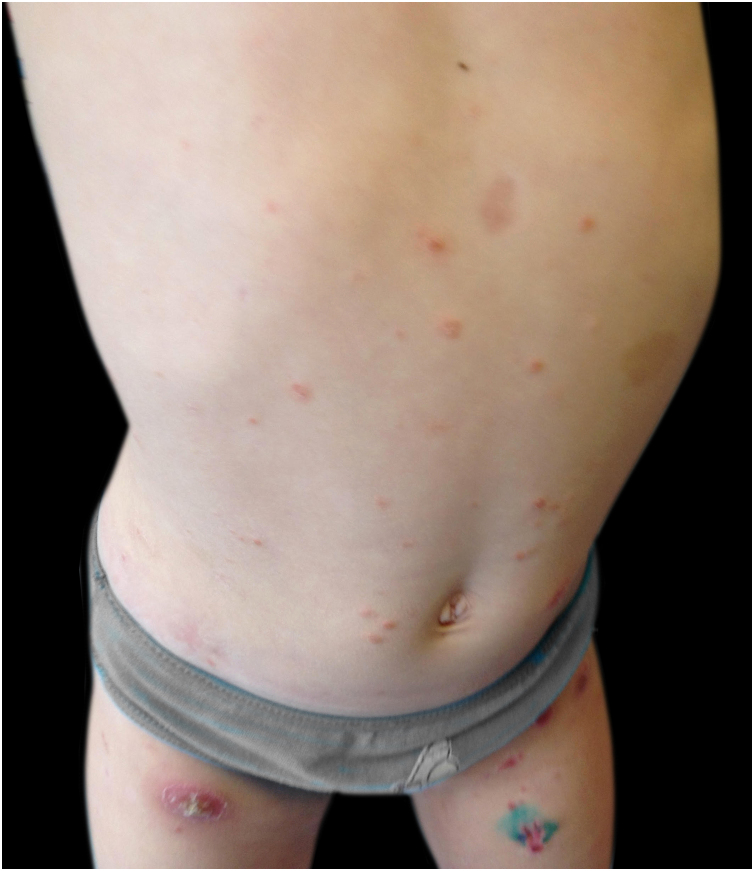
Erythematoviolaceous papules and nodules with slight desquamation on the abdomen and lower limbs.

**Figure 3:**
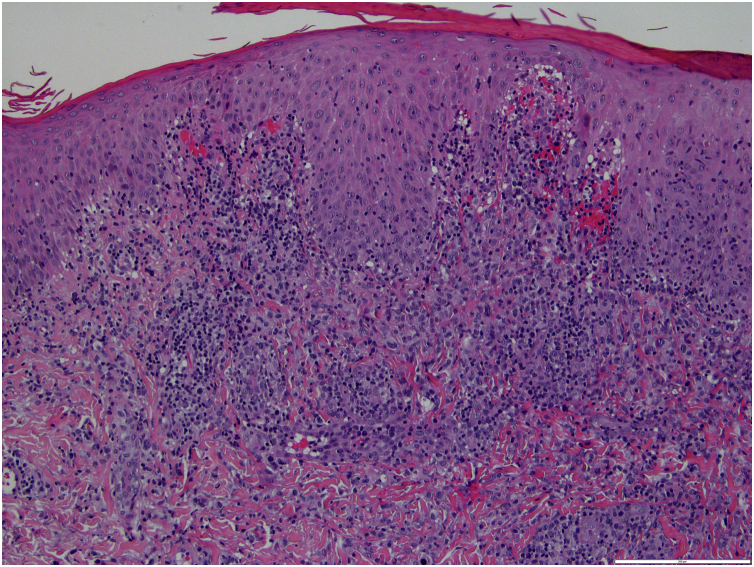
Multifocal dermal infiltrate of small lymphocytes, some plasma cells and histiocytes, as well as several lymphoid cells with amphophilic cytoplasm and big nucleus with prominent nucleolus (Hematoxylin & eosin, ×100).

Histopathology showed a multifocal dermal infiltrate of small lymphocytes, some plasma cells, and histiocytes, as well as several lymphoid cells with amphophilic cytoplasm, a big nucleus with the prominent nucleolus, and positivity to CD30, consistent with a type A lymphomatoid papulosis (LyP). The patient was treated with clobetasol propionate 0.5 mg/g ointment twice daily with progressive clearance of the lesions and has been symptom-free for the last four years.

LyP is a rare lymphoproliferative disorder that is included in the spectrum of primary cutaneous CD30-positive T-cell lymphoproliferative disorders by the World Health Organization classification of lymphoid tissue tumors.^1^ It slightly affects more men than women and usually presents with papulonodular lesions on the limbs and trunk, sometimes with necrosis, developing in crops.^2^, ^3^ Pruritus is a common feature.^2^

LyP is a recurrent disease, with a typical waning and waxing course of self-healing lesions.^4^ Lyp is seen most commonly in adults, although it has been suggested that the spontaneous resolution of lesions and their limited number in pediatric age may underestimate its frequency.^2^ In fact, the clinical presentation and course of LyP in childhood differs very little from the adult form.^2^ Albeit being rare among pediatric patients, LyP is one of the two most prevalent cutaneous lymphoproliferative diseases at this age, after mycosis fungoides.^4^

Over the years, the histopathological classification of LyP has been a changing scenario. Currently, five classically recognized histopathological subtypes have been defined, which vary based on the predominant cell type and tropism: type A (mixed cellular), type B (epidermotropic), type C (cohesive infiltrate), type D (epidermotropism), and type E (angiocentric and angiodestructive).^1^, ^3^, ^4^ The histopathological characterization has no prognostic value nor guides the treatment algorithm. The most common pathologic subtype of LyP is type A (also observed in the present study’s patient), which is characterized by a wedge-shaped dense dermal perivascular lymphoid infiltrate with large atypical CD30-positive cells with Reed-Sternberg appearance.^2^, ^5^

The differential diagnosis of LyP includes both forms of pityriasis lichenoides (pityriasis lichenoides et varioliformis acuta ‒ PLEVA ‒ and pityriasis lichenoides chronica ‒ PLC), primary cutaneous anaplastic large cell lymphoma (ALCL), a papular variant of mycosis fungoides and arthropod bites. More remotely, but mainly in childhood, other diagnoses such as scabies, pityriasis rosea, guttate psoriasis, and eczema should be considered.^1^, ^2^, ^3^ Some clinical tips may help in differentiating these diseases. For instance, as opposed to LyP, primary cutaneous ALCL presents usually as a solitary lesion and PLEVA presents with smaller lesions that usually do not show a waxing and waning nature and heal without scarring.^3^

In the absence of molecular or immunohistochemical criteria to predict LyP evolution, the clinicopathological correlation and long-term follow-up are indispensable. In fact, although LyP has classically a benign and indolent clinical course, some cases progress to other types of lymphoma, such as primary cutaneous ALCL, mycosis fungoides, or Hodgkin disease.^1^, ^4^ Fortunately, in pediatric patients, progression to lymphoma is exceptional.^2^ Moreover, some factors such as association with viral infections, atopic dermatitis, skin eosinophilic infiltration, and spontaneous complete remission strongly suggest that pediatric LyP could be considered a reactional disease rather than a neoplastic disorder.^2^

The long-term prognosis of pediatric LyP is generally considered to be good, with a 10-year disease-related survival rate of 100%.^1^, ^2^, ^3^ First-line treatment generally comprises therapeutic abstention or skin-directed modalities such as topical steroids.^1^, ^3^ Methotrexate and phototherapy are also commonly prescribed.^5^ There is no decreased risk of lymphoma in patients submitted to treatment, whereby the “wait and see” approach is also often applied.^1^, ^5^

## Financial support

None declared.

## Authors’ contributions

Nuno Gomes: Conception and design of the study, or acquisition of data, or analysis and interpretation of data, drafting the article or revising it critically for important intellectual content, final approval of the version to be submitted.

Ana Nogueira: Conception and design of the study, or acquisition of data, or analysis and interpretation of data, drafting the article or revising it critically for important intellectual content, final approval of the version to be submitted

Roberto Silva: Conception and design of the study, or acquisition of data, or analysis and interpretation of data, drafting the article or revising it critically for important intellectual content, final approval of the version to be submitted

Filomena Azevedo: Conception and design of the study, or acquisition of data, or analysis and interpretation of data, drafting the article or revising it critically for important intellectual content, final approval of the version to be submitted

## Conflicts of interest

None declared.
